# Maladaptive Perfectionism and Internet Addiction among Chinese College Students: A Moderated Mediation Model of Depression and Gender

**DOI:** 10.3390/ijerph18052748

**Published:** 2021-03-09

**Authors:** Wenjie Yang, Nobuaki Morita, Zhijuan Zuo, Kyoko Kawaida, Yasukazu Ogai, Tamaki Saito, Wenyan Hu

**Affiliations:** 1 The Mental Health Center, Yunnan University, Kunming 650091, China; ywj132278@gmail.com (W.Y.); zuozhijuan@sina.com (Z.Z.); 2 Department of Social Psychiatry and Mental Health, Faculty of Medicine, University of Tsukuba, Tsukuba 3050006, Japan; nobuakim@nifty.com (N.M.); kyoko-t@ca3.so-net.ne.jp (K.K.); ogai.ys@md.tsukuba.ac.jp (Y.O.); hhd02063@gmail.com (T.S.); 3 Mental Health Education Center for College Students, Zhejiang Gongshang University, Hangzhou 310018, China

**Keywords:** maladaptive perfectionism, internet addiction, depression, gender, college student

## Abstract

The association between perfectionism and addictive behaviors has been examined in previous literature; however, few pieces of research have investigated the mediating and moderating mechanisms underlying this relationship. Using a sample of 2016 Chinese college students, the present study examined the mediator of depression between maladaptive perfectionism and Internet addiction and the moderator of gender in such associations. The findings indicated that maladaptive perfectionism was directly related to students’ Internet addiction and indirectly predicted students’ Internet addiction via the mediator of depression. Gender moderated the direct effect, rather than the indirect effect, of maladaptive perfectionism on Internet addiction. Even though males reported a lower score on Internet addiction compared to females, the effect of maladaptive perfectionism on Internet addiction was stronger for males than for females. These findings revealed the psychological mechanisms from perfectionism to Internet addiction, which contributed to the theoretical development in addiction research and provided implications for interventions to reduce Internet addiction among Chinese college students.

## 1. Introduction

With the rapid popularization of smartphones, Internet accessibility has become widespread. Internet users reached 3.9 billion worldwide, and the rate of Internet use increased from 7.7% to 45.3% between 2005 and 2018 in developing countries [1]. According to recent data released by the China Internet Network Information Center (CNNIC), there were around 989 million Internet users in China, more than one-fourth of whom were students [2].

Currently, people’s daily life has become inseparable from the Internet, especially for college students who have grown up in the Internet era [3]. The Internet was widely used by college students for multiple purposes such as learning, entertainment, and communication. The increased availability and regular access to the Internet might facilitate excessive Internet use, which was proven to be a potential risk factor for the development of Internet addiction (IA) [3,4].

While Internet use is generally beneficial for peoples’ life [5,6], uncontrolled Internet use and IA are highly problematic, which might damage individuals’ well-being and lead to a series of adverse developmental outcomes [7,8]. A meta-analysis covering 70 studies found that the overall prevalence of IA among Chinese college students was 11.3% [9]. Considering the increasing prevalence of IA, rigorous research is needed to identify the risk factors of IA so as to provide implications for prevention and intervention programs to reduce IA among college students. To this end, the present study aims to investigate the associations among maladaptive perfectionism, depression, and IA in a sample of Chinese college students.

### 1.1. Maladaptive Perfectionism and Internet Addiction

Perfectionism is a personality trait that involves high standards of performance accompanied by a tendency for overly critical self-evaluation [10]. The conceptualization of perfectionism includes both adaptive and maladaptive components, namely perfectionistic strivings and perfectionistic concerns [11,12]. The former could help individuals to perform well, feel satisfied, and achieve goals, which has been suggested to be related to positive outcomes [13,14]. However, perfectionism could also be maladaptive when individuals focus on the discrepancy between ambitious standards and their actual performance [15,16]. Such maladaptive perfectionism has been linked to many negative outcomes, one of which is addictive behavior. Previous research has identified the predictive effect of maladaptive perfectionism on various forms of addictive behaviors, such as alcohol problems [17], disordered gambling [18], exercise addiction [19], and work addiction [20].

With the development of technology, research on technological addictions, such as IA and smartphone addiction, has increased recently. Previous studies also attempted to explore how maladaptive perfectionism impacted these forms of addictions. For example, Yoon, Kim, and Han (2017) found that pursuing overwhelming demands proposed by others significantly predicted social networking service (SNS) addiction [21]. Lehmann and Konstam (2011) showed that individuals with higher levels of maladaptive perfectionism were more likely to engage in Internet use [22]. Based on a sample of Chinese college students, Lingkai and Huashan (2015) found that “concern over mistakes” and “parents’ expectation,” as two components of maladaptive perfectionism, had a positive effect on IA. Accordingly, we assumed that maladaptive perfectionism could be a risk factor for IA [23].

### 1.2. Depression as a Mediator

The general strain theory has been widely used for analyzing addictive behaviors in recent years [24,25,26]. As suggested by this theory, strains would lead to negative emotions and psychological distress, which, in turn, would increase individuals’ risk to conduct problematic behaviors [24,27]. Accordingly, depression could be a potential mediating mechanism that links strain and IA. However, this theory attributed the causes of IA to the strains in the external social system [28], while neglecting the potential impact of personality traits on addictive behaviors. The Interaction of Person-Affect-Cognition-Execution (I-PACE) model is a recently proposed model that describes the psychological processes underlying the development of IA [29,30]. According to this theory, Internet-use disorders are considered to be the consequence of the interactions between individuals’ predisposing factors (e.g., personality) and affective responses. This theory could provide a theoretical foundation for the current study, in which we aim to understand how a personality factor (perfectionism) influences IA and to reveal a possible mediating effect of depression in the link between maladaptive perfectionism and IA.

On the one hand, perfectionism was validated as a risk factor for multiple psychological disorders [31,32]. Theoretically, the existential model of perfectionism and depressive symptoms (EMPDS) could explain why perfectionism is predictive of psychological distress [33]. This theory states that it is difficult for perfectionists to perceive their lives as purposeful, satisfying, and meaningful, making them risky for psychological disorders such as depression [33]. In line with this perspective, empirical studies have identified that maladaptive perfectionism was positively predictive of various psychological maladjustments such as depression, anxiety, and stress [34,35,36].

On the other hand, depression was also proved to be a psychological risk factor for IA. As indicated by the self-medication model [37], individuals might use the Internet to escape negative emotions and cope with emotional difficulties in reality [38,39,40]. Accordingly, in the present era, individuals have convenient access to the Internet and may be accustomed to relying on online activities to avoid negative affective states like depression, thereby possibly leading to IA [41,42,43]. Supporting the above theoretical statements, the findings of empirical studies yield that there was a significantly positive association between psychological distress and IA [39,44,45,46]. To be specific, Liang et al. (2016) found that higher levels of depression were associated with higher Internet-addictive behaviors [47]. Brailovskaia, Velten, and Margaf (2019) demonstrated that people with depressive symptoms were likely to use Facebook intensively to find relief and recover from stress [48]. Matar Boumosleh and Jaalouk (2017) also found that after controlling for social demographic variables, depression and anxiety positively predicted smartphone addiction of college students [49]. Taken together, maladaptive perfectionism might lead to a high level of depression, which in turn, might positively predict the level of IA. Thus, it was rational to assume that depression could be a potential mediator that linked the association between maladaptive perfectionism and IA.

### 1.3. Gender as a Moderator

Previous studies have examined the gender differences in IA. In general, most of the existing research indicated that the rate and level of IA were significantly higher among males in comparison to females [50]. This pattern has been observed in various countries such as China [51], India [50], Turkish [52], and Italy [53]. As suggested by Anand et al. [50], in most social contexts all over the world, there were fewer constraints on males compared to females in the process of socialization. Thus, it was possible that males were more likely than females to participate in recreational online activities such as online chatting, gaming, and gambling [50,54], thereby increasing their probability of developing IA.

Moreover, prior literature also suggested that gender might play a moderating role on IA, indicating that the strength of the associations between the risk factor and IA were significantly different between males and females. For instance, Liang et al. (2016) reported that the relationship between depression and IA was gender dependent and that males and females exhibited different behavioral patterns and motivations of Internet usage [47]. The prediction of depression on Internet addiction was higher in boys than in girls [47]. Similar findings have been also reported by Chen et al. [55], which revealed that the effect of conformity on smartphone addiction was stronger for boys than for girls. Based on this evidence, there would be differences in the levels of IA and on the strength of the associations among perfectionism, depression, and IA between males and females. Thus, we hypothesized that the mechanisms linking perfectionism with IA might vary as a function of gender.

### 1.4. The Present Study

**Figure 1:**
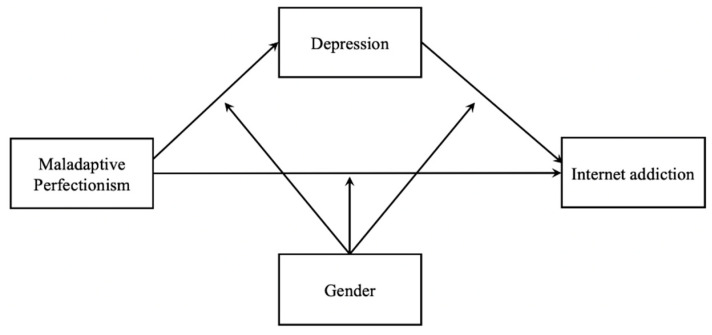
Conceptual framework.

Some research gaps existed in the existing literature in the field of IA. Firstly, previous research has primarily focused on the influence of social determinants on IA, and little is known about how individual factors such as personality and psychological distress related to IA. Additionally, among the limited number of literature on IA, few studies have examined the mediation process and moderation effects. Moreover, previous research has been mainly conducted in western countries, and the empirical evidence is relatively rare in mainland China. Considering that perfectionism differed across individualistic and collectivistic cultures [56], it was necessary to examine whether the relevant theories could be supported and whether prior findings could be replicated in the Chinese context. Based on the above review, the present study established a moderated mediation model to examine the process by which maladaptive perfectionism predicted IA, and how the mechanisms differed by gender. The proposed model was shown in Figure 1. We proposed the following research hypotheses.

**Hypothesis** **1** **(H1).** *Maladaptive perfectionism would have a direct effect on IA.*

**Hypothesis** **2** **(H2).** *Maladaptive perfectionism would have an indirect effect on IA via the mediator of depression. Specifically, maladaptive perfectionism would positively predict depression, which in turn, would increase the level of IA.*

**Hypothesis** **3** **(H3).** *Gender would moderate the direct and indirect effects of maladaptive perfectionism on IA. The strength of the associations would be different between males and females.*

## 2. Methods

### 2.1. Participants and Procedure

We conducted a cross-sectional online survey to collect data from a university in southwest China. The QR code of the survey was displayed on the screen, and the students could scan and deposit it on their smartphones to complete the survey. The participants were provided informed consent forms before they clicked the survey link. Completing the questionnaire and clicking submit was taken as implied consent. The questionnaire took 10 to 15 min to complete. The implementation of the study and the analysis of the data were approved by the Ethics Review Committee of the university where the authors were affiliated. A total of 2238 college students participated in the survey, and finally, we obtained 2016 (mean age = 18.3, SD = 0.80, 43.1% male) valid questionnaires, with a valid response rate of 90.1%. The participants were from 34 provinces of mainland China with various backgrounds. The demographic characteristics of the sample are shown in Table 1.

### 2.2. Measures

#### 2.2.1. Internet Addiction

To assess the participants’ IA levels, we implemented the Chen Internet Addiction Scale-revised (CIAS-R), which was developed particularly for Chinese respondents [57]. The CIAS-R is a 26-item questionnaire that uses a 4-point Likert scale (“1 = does not match my experience at all” to “4 = definitely matches my experience”). Responses across the items are then summed, with higher scores indicating higher levels of IA. The Cronbach’s alpha in this study was 0.94.

#### 2.2.2. Maladaptive Perfectionism

We adopted the Frost Multidimensional Perfectionism Scale (FMPS) [10] to measure the participants’ maladaptive perfectionism. This scale was translated and revised by Zi and Zhou [58] specifically for Chinese college students. The FMPS contains 27 items with 5 dimensions of perfectionism: Concern over Mistakes (CM), Doubts about Actions (DA), Personal Standards (PS), Parental Expectations (PE), and Organization (OR). Among them, Organization belongs to adaptive perfectionism, while the other 4 dimensions belong to maladaptive perfectionism. A 5-point Likert scale (“1 = strongly disagree” to “5 = strongly agree”) was adopted. We summed up the scores of the CM, DA, PS, and PE, with a higher score indicating a higher maladaptive perfectionism. The Cronbach’s alpha in this study was 0.88.

#### 2.2.3. Depression

We implemented the Chinese version of the Self-Rating Depression Scale (SDS) [59] to assess the mental health status of the participants. It contains 20 items with a 4-point Likert scale (“1 = not at all” to “4 = very much”), with higher scores indicating more severe depressive symptoms. The Cronbach’s alpha in this study was 0.85.

### 2.3. Data Analysis

We performed a descriptive analysis for the characteristics of the research sample and the bivariate correlations among the main variables. SPSS macro PROCESS [60], which was specifically developed for assessing complex models, including both mediating and moderating effects, was utilized to test the hypothetical model of the current study. We first tested the mediating effect of depression between maladaptive perfectionism and IA. The bias-corrected percentile bootstrap method was adopted to determine the indirect effect. A significant mediation effect was determined when the 95% bias-corrected bootstrap CI did not contain zero [60]. Then, we included gender as a moderator into the above mediation model to further test the moderated mediation effect. Participants’ age, numbers of siblings, smartphone usage time, and monthly consumption were controlled as covariates in the analysis.

## 3. Results

### 3.1. Preliminary Analyses

The results of descriptive statistics and correlation analysis are shown in Table 2. We found that maladaptive perfectionism was positively correlated with depression (r = 0.330, *p* < 0.01) and IA (r = 0.295, *p* < 0.01). There was also a positive correlation between depression and IA (r = 0.426, *p* < 0.01).

The results of the independent-sample *t* test are presented in Table 3, which demonstrate the gender differences in the independent (perfectionism), dependent (IA), and mediating variables (depression). As shown, males reported higher levels of perfectionism than females (60.695 > 58.663, t = 3.278, *p* < 0.001), whereas females reported higher levels of depression (35.497 > 34.631, t = –2.413, *p* < 0.05) and IA (51.665 > 49.552, t = –3.536, *p* < 0.001) than males.

### 3.2. Testing for Mediation Effect

The results of the mediating effect of depression are shown in Table 4. As expected, the direct effect of maladaptive perfectionism on IA was significant (b = 0.165, *p* < 0.001). We also found that maladaptive perfectionism positively predicted depression (b = 0.188, *p* < 0.001), which further increased the level of IA (b = 0.562, *p* < 0.001). The results of bootstrapping indicated that perfectionism had a significant indirect effect on IA via the mediator of depression (b = 0.105, SE = 0.010, 95% CI = [0.087, 0.126]).

Considering the effects of covariates, we found that the number of siblings was significantly related to IA (b = 1.750, *p* < 0.01), which indicated that students with siblings were more likely to be addicted to the Internet compared with the only child in the family. Students’ smartphone usage time also positively predicted students’ IA (b = 4.474, *p* < 0.001). Other control variables, including age (b = 0.102, *p* > 0.05) and monthly consumption (b = −0.279, *p* > 0.05) were not significantly associated with IA among college students.

### 3.3. Testing for Moderated Mediation

**Figure 2:**
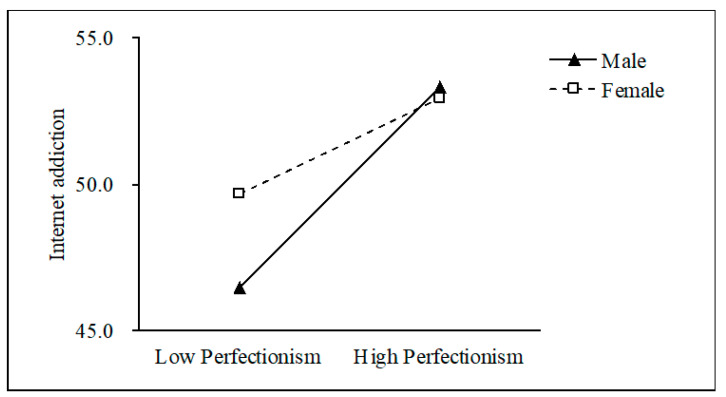
Gender as a moderator of the relationship between perfectionism and Internet addiction.

Table 5 presents the results of the moderated mediation model. As shown in this table, the effect of the interaction term (Perfectionism × Gender) was not significant for depression (b = 0.006, *p* > 0.05), while it was significant for IA (b = −0.132, *p* < 0.01). These results indicated that gender did not moderate the path from perfectionism to depression, but it significantly moderated the association between perfectionism and IA. Moreover, there was not a significant association between the interaction term (Depression × Gender) and IA (b = −0.056, *p* > 0.05), which demonstrated that gender did not play a moderating role in the association between depression and IA. For descriptive purposes, we plotted predicted IA against perfectionism, separately for males and females (shown in Figure 2). Simple slope tests showed that the positive effect of perfectionism on IA was stronger for males (b_simple_ = 0.250, *p* < 0.001) than for females (b_simple_ = 0.118, *p* < 0.001). These results indicated that the direct effect of perfectionism on IA was moderated by gender.

## 4. Discussion

Using a sample of Chinese college students, this study explored the mediating effect of depression between perfectionism and IA and validated whether this effect varied by gender. Our findings indicated that perfectionism increased the level of depression in college students and further increased their risk of developing IA. Perfectionism had a stronger association with IA among males relative to females. The main findings can be discussed as follows.

### 4.1. Self-Medication Process

In recent years, research on how perfectionism influenced addictive behaviors has increased, but most of the studies were conducted in western countries. We found that perfectionism was directly related to IA, and it was consistent with previous research that illustrated that individuals with higher levels of maladaptive perfectionism were more likely to use the Internet problematically and develop IA [22,61]. More importantly, this study revealed the mediating effect of depression. Our findings supported the existential model of perfectionism and depressive symptoms [33] and the self-medication theory [37], and they were in line with previous findings that showed positive associations between perfectionism and psychological distress [32,34] and associations between depression and IA [47,48]. We provided rational explanations for these findings in the Chinese context.

There are differences in the effect of perfectionism across collectivistic and individualistic cultures [56,62]. As validated in previous research, college students from a collectivism-oriented country (e.g., Japan) reported lower levels of self-oriented perfectionism (i.e., to be a perfectionist to meet self-requirement) and higher levels of socially prescribed perfectionism (i.e., to be a perfectionist to meet others’ standards) than those from individualism-oriented societies such as the US and the UK [63,64,65]. Similar to Japan, China is a country dominated by a collectivistic culture, in which people hope to meet the demands of others to maintain harmonious interpersonal relationships. Under such cultural backgrounds, perfectionists tend to perceive external pressures to perform well, which places them at risk of depressive symptoms.

In addition, the social expectation model states that children may internalize high standards and expectations from their parents and develop perfectionism [66]. Nowadays, most college students are the only child in Chinese families due to China’s one-child policy implemented in 1978. Influenced by Confucius culture, Chinese parents may adopt authoritative parenting and set high standards for their children. Being raised in such contexts, Chinese college students might pursue unachievable goals derived from social expectations. If they failed to achieve these goals, it may be difficult for them to feel satisfied and realize self-worth, which further places them at risk of depressive symptoms [67,68]. Moreover, almost every Chinese university campus is currently covered by the Internet. Based on the notion of “self-medication” [37], easy access to the Internet and the prevalent use of electronic devices such as laptops and smartphones provide opportunities for college students to surf the Internet for pleasure and use it as a strategy to release psychological pressure and escape negative emotions, which increase the probability of developing IA.

### 4.2. Gender Differences

In terms of gender differences, our findings suggested that females reported a higher level of IA than males. This was inconsistent with most prior studies, which showed that male students were more susceptible to IA relative to females [9]. The inconsistency may arise from the coverages of Internet use employed by the studies. In our study, the use of smartphones was included in the instruction of the measurement of IA. A previous study showed that compared with males, female students were more likely to be addicted to smartphones [69], which might explain the current finding that females showed a higher level of IA than males. In addition, China’s e-commerce has developed rapidly in recent years, and online shopping in mobile terminals is the most common consumption pattern among college students. In relation to males, female students showed a higher interest in online shopping. Therefore, after including smartphone use in the measurement of Internet addiction, our research indicated an inconsistent finding with previous literature, which only focused on Internet use on the computer.

Furthermore, we found that gender moderated the relationship between perfectionism and Internet addiction, with the strength of association being stronger for males than females. Our results showed that males reported a higher perfectionism score than females, which was consistent with the results obtained by prior literature [70,71]. This may be attributed to the higher social expectation toward males under the traditional gender role division in China [72]. The results of our study also suggested that males were more likely to develop IA than females in the presence of perfectionism, and this finding could be explained by the different purposes of Internet usage between males and females [73]. In particular, males tend to use the Internet for entertainment such as playing online games in comparison to females who surf the Internet more for social activities such as online communication and social interaction [47,73,74]. Compared with social activities, recreational activities are more likely to attract people to indulge in Internet use to avoid high expectations and their stressful reality. Previous studies have also shown that females may use positive cognitive patterns more than males to reappraise negative emotions caused by perfectionism [75,76] and were therefore less likely to use IA to cope with negative emotions. The gender differences in emotional coping strategies could also provide an explanation for the finding that boys experienced a faster increase in IA than girls in the presence of perfectionism.

### 4.3. Contributions and Implications

With respect to theoretical contributions, one strength of the present study was that we validated western-originated theories (e.g., self-medication theory) in China, which provided cross-cultural empirical evidence for the applicability of these theories in the Chinese cultural context.

Additionally, even though the technological forms of addiction have received researchers’ attention recently, past research offered few evidence on how perfectionism affected IA. The present study deepened the understanding of the individual risk factors of IA by revealing how it was related to personality and psychological factors. To our knowledge, this study is the first to establish a moderated mediation model that examined the associations among perfectionism, depression, and IA in Chinese college students. The findings extended previous literature by revealing the mediating mechanism through which perfectionism was related to IA and the moderating role which explained the gender differences in the mechanisms from perfectionism to IA.

Although gender differences on IA have been described in prior studies, there was a lack of research explicitly examining the role of gender when investigating the relationship between perfectionism and IA, as in the present study. In this aspect, our research renewed empirical insights into the gender differences of IA. With the development of science and technology and dramatic social changes, people’s lifestyle and Internet usage patterns have undergone rapid changes in China. Based on recently collected data, our research obtained certain unexpected results that were inconsistent with prior literature and provided a rational explanation by taking the current Chinese social background into consideration. Such findings also provide directions for future research designs, such as the need to adapt IA measurements following the trend of technology development.

The findings of the current study also had important practical implications. We found that perfectionism was positively predictive of IA, thus this personality trait should be considered in the design of targeted interventions to prevent IA. Perfectionism is assumed to be constructed during the process of socialization and is considered to be stable in adults as an enduring personality trait [77]. Projects for reducing maladaptive perfectionism should target children and their social systems such as families and schools because childhood is a critical stage for the development of perfectionism traits [78]. In addition, the mediating role of depression between perfectionism and IA inspired that mental health education for college students is critical to preventing IA. Furthermore, therapeutic interventions could also be designed to improve students’ ability to cope with negative emotions to block the process of self-medication. An intervention model that could be adapted and implemented in China is the Stress Temperament Coaching [79], which was proven to be effective in alleviating psychological distress by promoting active coping. Finally, the significant moderating role of gender emphasized gender-sensitive approaches to prevent IA in Chinese college students.

### 4.4. Limitation and Future Directions

Despite the vital contributions of this study, there were several limitations that should not be neglected. Firstly, because of the study’s cross-sectional design, it could not determine any causal relationships. Thus, longitudinal studies should be conducted in the future to validate the current findings. Secondly, data of this study were collected from a single university. Despite the relatively large and diverse sample size, the participants may not be representative of the overall population of Chinese college students, and the research findings should be generalized with caution. Moreover, owing to the use of an online self-rating measurement, our participants might report fewer Internet-addictive behaviors owing to the social desirability effect. Future research could use multiple sources of data to improve the research validity. Finally, we did not clarify the specific contents of IA in this study, such as game addiction or social networking service (SNS) addiction. It could be further addressed by future research to examine the specific forms of IA and to obtain convincing results, especially when explaining gender differences.

## 5. Conclusions

Overall, the findings suggested that depression was a psychological mechanism through which maladaptive perfectionism was associated with Internet addiction among Chinese college students. In addition, we found that males were more likely to develop Internet addiction in the presence of perfectionism compared with females. The findings extended the applicability of self-medication theory in the explanation of Internet addiction. This study also had important clinical implications for the prevention and intervention programs to reduce Internet addiction among Chinese college students.

## Figures and Tables

**Table 1:** Demographic characteristics of participants (*n* = 2016).

Variables	*N* (%)
Age	M = 18.3 (SD = 0.8)
Gender	
Male	868 (43.1)
Female	1148 (56.9)
Only child	
Yes	848 (42.1)
No	1168 (57.9)
Smartphone daily usage time	
Under 4 h	845 (41.9)
4–8 h	1008 (50.0)
Over 8 h	163 (8.1)
Monthly consumption	
Under 1200 yuan	1148 (56.9)
1200–1999 yuan	753 (37.4)
Over 2000 yuan	115 (5.7)

**Table 2:** Descriptive statistics and bivariate correlations of key variables.

Variable	M	SD	1	2	3
1. Maladaptive perfectionism	59.538	13.815	1		
2. Depression	35.124	7.980	0.330 **	1	
3. Internet addiction	50.755	13.181	0.295 **	0.426 **	1

** *p* < 0.01 (2-tailed).

**Table 3:** Description and comparison of key variables by gender.

Variable	Gender	M	SD	t
Maladaptive perfectionism	Male	60.695	13.329	3.278 ***
	Female	58.663	14.114	
Depression	Male	34.631	8.163	−2.413 *
	Female	35.497	7.821	
Internet addiction	Male	49.552	13.716	−3.536 ***
	Female	51.665	12.691	

* *p* < 0.05, *** *p* < 0.001.

**Table 4:** Result of mediation analysis.

	Model 1: Depression				Model 2: Internet Addiction			
	B	SE	t	*p*	B	SE	t	*p*
Age	0.102	0.224	0.455	0.649	−0.083	0.341	−0.242	0.809
Only child	0.706	0.349	2.026	0.043	1.750	0.531	3.297	0.001
Smartphone usage time	1.402	0.271	5.171	<0.001	4.474	0.415	10.782	<0.001
Monthly consumption	−0.279	0.286	−0.973	0.331	0.466	0.435	1.070	0.285
Perfectionism	0.188	0.012	15.525	<0.001	0.165	0.019	8.496	<0.001
Depression					0.562	0.034	16.576	<0.001
R^2^	0.123				0.257			
F	56.444 ***				116.000 ***			

*** *p* < 0.001.

**Table 5:** Result of moderated mediation analysis.

	Model 1: Depression				Model 2: Internet Addiction			
	B	SE	t	*p*	B	SE	t	*p*
Age	0.148	0.224	0.659	0.510	−0.019	0.340	−0.055	0.956
Only child	0.617	0.349	1.769	0.077	1.658	0.530	3.129	0.002
Smartphone usage time	1.326	0.272	4.884	<0.001	4.420	0.414	10.666	<0.001
Monthly consumption	−0.330	0.286	−1.153	0.249	0.387	0.434	0.893	0.372
Perfectionism	0.190	0.012	15.727	<0.001	0.174	0.020	8.957	<0.001
Gender	1.100	0.340	3.235	0.001	1.398	0.517	2.703	0.007
Perfectionism × Gender	0.006	0.025	0.260	0.795	−0.132	0.040	−3.345	0.001
Depression					0.555	0.034	16.357	<0.001
Depression × Gender					−0.056	0.068	−0.824	0.410
R^2^	0.128				0.266			
F	42.001 ***				80.549 ***			

*** *p* < 0.001.

## Data Availability

No new data were created or analyzed in this study. Data sharing is not applicable to this article.
